# Protective role of cytosolic prion protein against virus infection in prion-infected cells

**DOI:** 10.1128/jvi.01262-24

**Published:** 2024-08-28

**Authors:** Hideyuki Hara, Junji Chida, Batzaya Batchuluun, Etsuhisa Takahashi, Hiroshi Kido, Suehiro Sakaguchi

**Affiliations:** 1 Division of Molecular Neurobiology, The Institute for Enzyme Research (KOSOKEN), Tokushima University, Tokushima, Japan; 2 Core Research Facility, Center for Infectious Disease Education and Research (CiDER), Osaka University, Osaka, Japan; 3 Division of Enzyme Chemistry, The Institute for Enzyme Research, Tokushima University (KOSOKEN), Tokushima, Japan; St. Jude Children's Research Hospital, Memphis, Tennessee, USA

**Keywords:** prion, prion protein, influenza A virus, NF-κB, NLRP3 inflammasome, necroptosis

## Abstract

Production of the amyloidogenic prion protein, PrP^Sc^, which forms infectious protein aggregates, or prions, is a key pathogenic event in prion diseases. Functional prion-like protein aggregations, such as the mitochondrial adaptor protein MAVS and the inflammasome component protein ASC, have been identified to play a protective role in viral infections in mammalian cells. In this study, to investigate if PrP^Sc^ could play a functional role against external stimuli, we infected prion-infected cells with a neurotropic influenza A virus strain, IAV/WSN. We found that prion-infected cells were highly resistant to IAV/WSN infection. In these cells, NF-κB nuclear translocation was disturbed; therefore, mitochondrial superoxide dismutase (mtSOD) expression was suppressed, and mitochondrial reactive oxygen species (mtROS) was increased. The elevated mtROS subsequently activated NLRP3 inflammasomes, leading to the suppression of IAV/WSN-induced necroptosis. We also found that prion-infected cells accumulated a portion of PrP molecules in the cytosol, and that the N-terminal potential nuclear translocation signal of PrP impeded NF-κB nuclear translocation. These results suggest that PrP^Sc^ might play a functional role in protection against viral infections by stimulating the NLRP3 inflammasome-dependent antivirus mechanism through the cytosolic PrP-mediated disturbance of NF-κB nuclear translocation, which leads to suppression of mtSOD expression and consequently upregulation of the NLRP3 inflammasome activator mtROS.

**IMPORTANCE:**

Cytosolic PrP has been detected in prion-infected cells and suggested to be involved in the neurotoxicity of prions. Here, we also detected cytosolic PrP in prion-infected cells. We further found that the nuclear translocation of NF-κB was disturbed in prion-infected cells and that the N-terminal potential nuclear translocation signal of PrP expressed in the cytosol disturbed the nuclear translocation of NF-κB. Thus, the N-terminal nuclear translocation signal of cytosolic PrP might play a role in prion neurotoxicity. Prion-like protein aggregates in other protein misfolding disorders, including Alzheimer's disease were reported to play a protective role against various environmental stimuli. We here showed that prion-infected cells were partially resistant to IAV/WSN infection due to the cytosolic PrP-mediated disturbance of the nuclear translocation of NF-κB, which consequently activated NLRP3 inflammasomes after IAV/WSN infection. It is thus possible that prions could also play a protective role in viral infections.

## INTRODUCTION

Prions are the causative agents of prion diseases, a group of neurodegenerative disorders, which include Creutzfeldt–Jakob disease in humans and scrapie and bovine spongiform encephalopathy in animals (1–4). They are self-templating protein aggregates consisting of the abnormally folded, amyloidogenic isoform of prion protein, designated PrP^Sc^ (1–4). PrP^Sc^ is produced by conformational conversion of the normal cellular isoform, PrP^C^, through a self-templating protein polymerization mechanism (1–4). PrP^C^ is a membrane glycoprotein tethered to the plasma membrane via a glycosylphosphatidylinositol (GPI) moiety and expressed most abundantly in the brain, particularly by neurons (5). We and others have shown that mice devoid of PrP^C^ (*Prnp^0/0^*) are resistant to prions, neither developing disease nor propagating PrP^Sc^ or prions in their brains after intracerebral inoculation with the prions (6–9), indicating that the conversion of PrP^C^ into PrP^Sc^ is a key pathogenic event in prion diseases.

Self-templating protein aggregates or prion-like protein aggregates have been also reported in yeasts (10–12). It still remains to be addressed whether or not yeast prions are toxic or beneficial to their hosts. Some investigators have demonstrated that some types of yeast prions could serve as an epigenetic mechanism to response to environmental stress (13–16). A structurally altered form of Ure2, a regulatory protein in the nitrogen metabolism, forms [URE3] prions and adapts the cells to nitrogen-poor conditions (17, 18). The [*PSI*^+^] prion, which is the protein aggregate of the translation termination factor Sup35, alters translational fidelity and thereby confers a proliferative advantage on the cells in fluctuating environments (19, 20). Mod5, a transfer RNA isopentenyltransferase, also forms [*MOD*^+^] prions in yeast, regulating the sterol biosynthetic pathway and protecting from anti-fungal agents (21). [Het-s] prion in the fungus *Podospora anserina* is involved in the heterokaryon incompatibility between [Het-s] and incompatible [Het-S] strains (22, 23). The heterokaryon incompatibility serves to prevent the horizontal transmission of mycoviruses and other parasites between strains by inducing cell death in fused incompatible strains (22, 23). However, other investigators reported that yeast cells harboring [URE3] and [*PSI*^+^] prions exhibited slow growth or cell death under the usual growth conditions, indicating that [URE3] and [*PSI*^+^] prions are toxic or lethal, not beneficial, to their host cells (24). Functional prion-like protein aggregates are also reported in mammalian cells (25, 26). Upon viral infections, cells form an inflammatory signaling platform called an inflammasome, which consists of apoptosis-associated speck-like protein containing a caspase activation and recruitment domain (ASC), the family members of nucleotide-binding oligomerization domain, leucine-rich repeat and pyrin domain containing protein (NLRP), and caspase-1 (27). Caspase-1 is then activated in the complex and cleaves pro-interleukin-1ß (pro-IL-1ß) and pro-IL-18 to convert them into functionally mature secretory forms, which eventually lead to activation of cellular protective mechanisms against viruses (27, 28). During inflammasome formation, ASC forms prion-like protein aggregates (25). The mitochondrial adaptor protein MAVS is also known to form prion-like aggregates in response to viral infection to elicit antiviral innate immunity (25, 26). However, it is unknown if mammalian PrP^Sc^prions could play a functional role against external stimuli.

In this study, we show that prion-infected cells were relatively highly resistant to infection with a neurotropic influenza A virus strain A/WSN/33 (H1N1) (hereafter referred to as IAV/WSN), suppressing IAV/WSN-induced necroptosis, by activating NLRP3 inflammasome. We then showed that, in prion-infected cells, the nuclear translocation of the transcription factor NF-κB was disturbed after IAV/WSN infection, therefore suppressing the gene expression of mitochondrial superoxide dismutase (SOD2), elevating mitochondrial reactive oxygen species (mtROS), and eventually activating the NLRP3 inflammasome. We also show that a portion of PrP molecules was accumulated in the cytosol of prion-infected cells and that the cytosol expression of the PrP N-terminal cryptic nuclear translocation signal interfered with the nuclear translocation of NF-κB in prion-uninfected cells. These results indicate that prion-infected cells could accumulate PrP molecules in the cytosol, thereby the nuclear translocation of NF-κB was disturbed through the PrP cryptic nuclear translocation signal and eventually NLRP3 inflammasome was activated to protect from IAV/WSN infection, suggesting that PrP^Sc^-prions might play a functional role in viral infections.

## RESULTS

### Prion-infected cells are partially resistant to IAV/WSN-induced cell death

**Fig 1:**
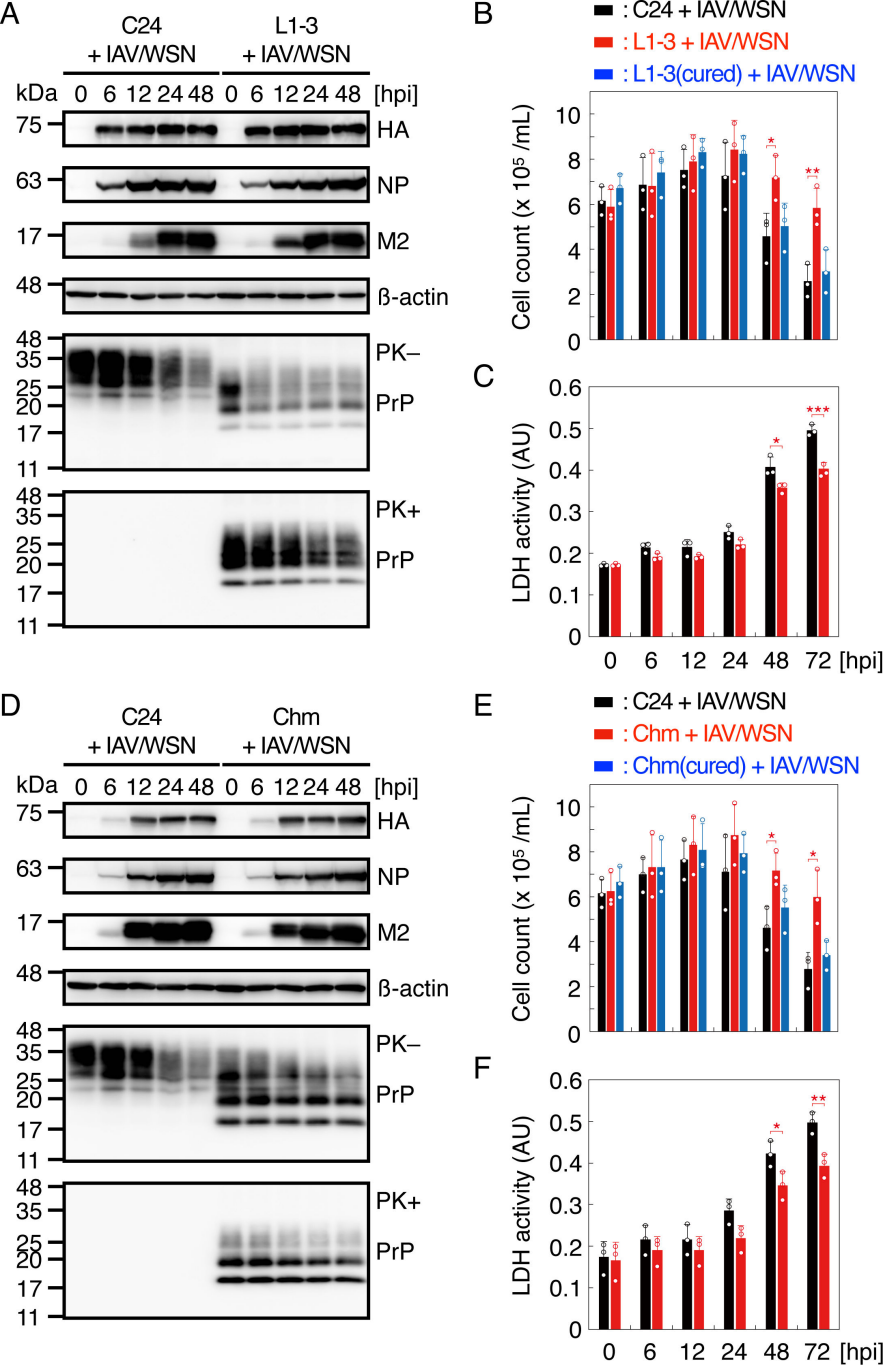
Prion-infected cells are partially resistant to IAV/WSN infection. (**A**) Western blotting of N2aC24 (**C24**) and N2aC24L1-3 (L1-3) cells at various time points after IAV/WSN infection for viral proteins, including HA, NP, and M2, an internal control β-actin, and PrP treated with (+) or without (-) PK. (**B**) The number of C24, L1-3, and curd L1-3 [L1-3(cured)] cells at various time points after IAV/WSN infection. (**C**) LDH activity in the medium of C24 and L1-3 cells at various time points after IAV/WSN infection. (**D**) Western blotting of C24 and N2aC24Chm (Chm) cells at various time points after IAV/WSN infection for viral proteins, HA, NP, and M2, an internal control β-actin, and PrP treated with (+) or without (-) PK. (**E**) The number of C24, Chem, and curd Chm [Chm(cured)] cells at various time points after IAV/WSN infection. (**F**) LDH activity in the medium of N2aC24 and Chm cells at various time points after IAV/WSN infection. hpi, hours post infection. Data are the mean ± standard deviation (SD) of three independent experiments. *, *P* < 0.05; **, *P* < 0.01; ***, *P* < 0.005.

To investigate if prion-infected cells are resistant to IAV/WSN infection, we infected IAV/WSN at a multiplicity of infection (MOI) of 1.0 into N2aC24L1-3 cells, which are exogenous mouse PrP^C^-overexpressing, 22L scrapie prion persistently infected cloned mouse neuroblastoma N2aC24 cells, and prion-uninfected N2aC24 cells as a control (29). Viral proteins, including HA, NP, and M2, were similarly produced in N2aC24 and N2aC24L1-3 cells after IAV/WSN infection (Fig. 1A). Consistent with IAV infection suppressing host protein synthesis (30), PrP^C^ in N2aC24 cells was reduced after IAV/WSN infection (Fig. 1A). IAV/WSN infection also reduced total PrP and PrP^Sc^ in N2aC24L1-3 cells (Fig. 1A). For cell viability assay, we additionally used cured N2aC24L1-3 cells, in which prions had been eliminated by treatment with SAF32 anti-PrP antibody (31), as a control. These cells died after IAV/WSN infection (Fig. 1B; Fig. S1A). However, cell viability was much higher for N2aC24L1-3 cells than for N2aC24 and cured N2aC24L1-3 cells after IAV/WSN infection (Fig. 1B; Fig. S1A). Moreover, cured N2aC24L1-3 cells exhibited similar cell viability to N2aC24 cells (Fig. 1B; Fig. S1A), suggesting that prion infection could confer N2aC24L1-3 cell resistance to IAV/WSN-induced cell death. Lactate dehydrogenase (LDH) was accordingly lower in the medium of N2aC24L1-3 cells than in N2aC24 cells after IAV/WSN infection (Fig. 1C). Similar results were obtained with N2aC24Chm cells, which are N2aC24 cells persistently infected with RML scrapie prions (Fig. 1D through F; Fig. S1B). These results indicate that prion-infected cells are more resistant to IAV/WSN-induced cell death than prion-uninfected cells.

### Prion-infected cells suppress IAV/WSN-induced necroptosis

**Fig 2:**
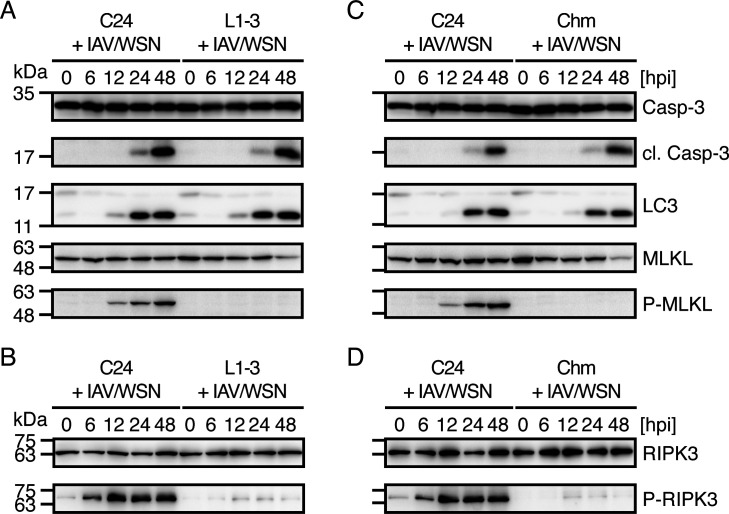
Necroptosis is suppressed in prion-infected cells after IAV/WSN infection. (**A**) Western blotting of N2aC24 (**C24**) and N2aC24L1-3 (L1-3) cells at various time points after IAV/WSN infection for caspase-3, cleaved caspase-3, LC3-II, MLKL, and phosphorylated MLKL (p-MLKL). (**B**) Western blotting of C24 and L1-3 cells at various time points after IAV/WSN infection for RIK3 and phosphorylated RIPK3 (p-RIPK3). (**C**) Western blotting of C24 and N2aC24Chm (Chm) cells at various time points after IAV/WSN infection for caspase-3, cleaved caspase-3, LC3-II, MLKL, and p-MLKL. (**D**) Western blotting of C24 and Chm cells at various time points after IAV/WSN infection for RIK3 and p-RIPK3. hpi, hours post infection.

To investigate which types of cell death are suppressed in prion-infected cells after IAV/WSN infection, we performed Western blotting for various cell death markers, including the apoptosis marker cleaved caspase-3, the autophagic cell death marker light chain 3-II (LC3-II), and the necroptotic marker phosphorylated mixed-lineage kinase domain-like pseudokinase (MLKL), in N2aC24 and N2aC24L1-3 cells after IAV/WSN infection. Cleaved caspase-3 and LC3-II were similarly increased in both cells after IAV/WSN infection (Fig. 2A). However, phosphorylated MLKL was markedly increased in N2aC24 cells, but barely detectable in N2aC24L1-3 cells, after IAV/WSN infection (Fig. 2A). Consistent with this, receptor-interacting kinase 3 (RIPK3), the kinase phosphorylating MLKL, was markedly phosphorylated in N2aC24 cells, but not in N2aC24L1-3 cells, after IAV/WSN infection, indicating that RIPK3 is activated in N2aC24 cells, but not in N2aC24L1-3 cells, after IAV/WSN infection (Fig. 2B). N2aC24Chm cells also showed similar results (Fig. 2C and D). These results suggest that necroptosis is specifically suppressed in prion-infected cells after IAV/WSN infection.

### Prion-infected cells activate NLRP3 inflammasome after IAV/WSN infection

**Fig 3:**
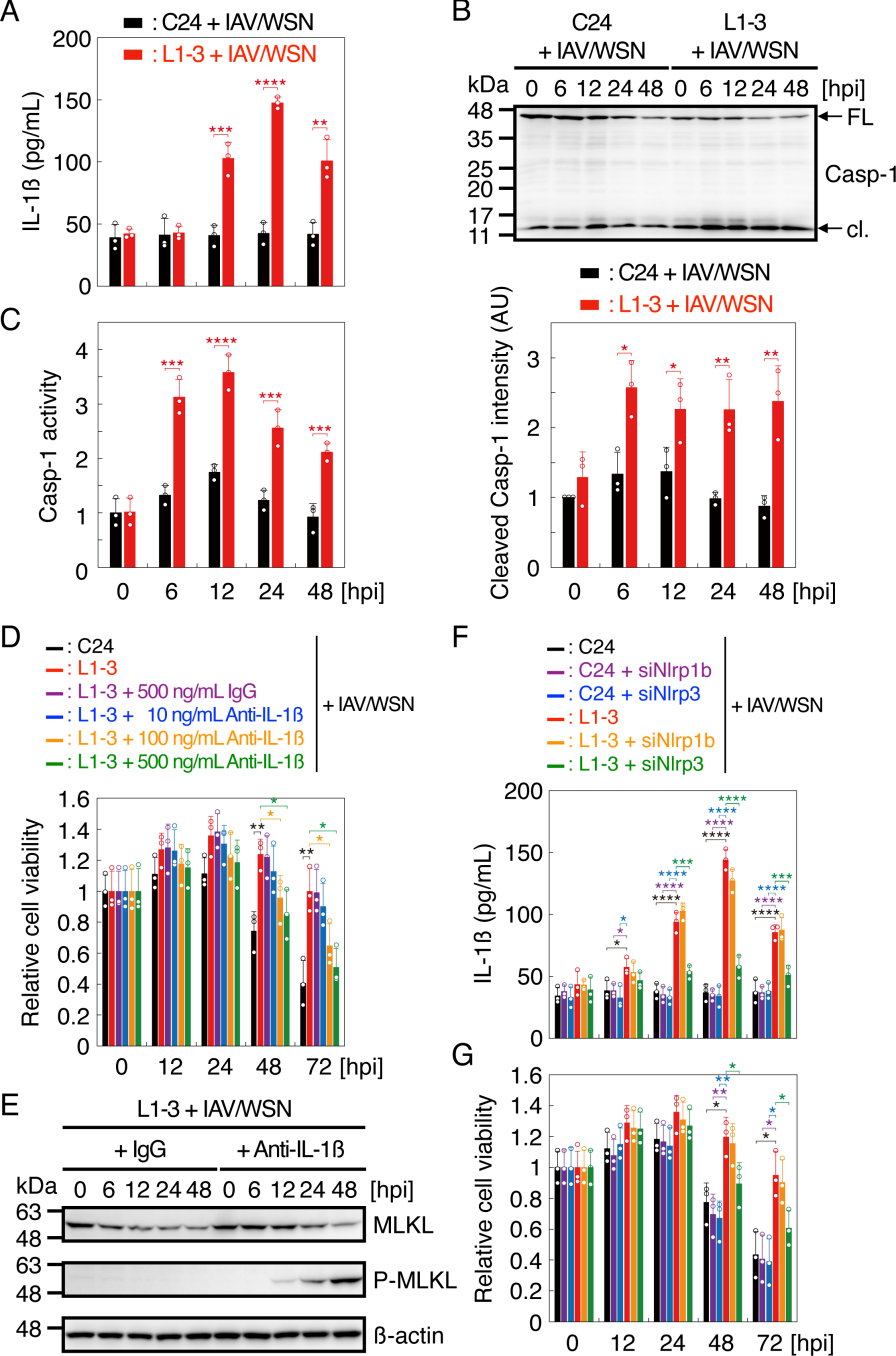
NLRP3 inflammasome is highly activated in prion-infected cells after IAV/WSN infection. (**A**) IL-1β in the medium of N2aC24 (**C24**) and N2aC24L1-3 (L1-3) cells at various time points after IAV/WSN infection. (**B**) Upper panel: Western blotting of C24 and L1-3 cells at various time points after IAV/WSN infection for caspase-1. Lower panel: Intensity comparison of cleaved caspase-1 in C24 and L1-3 cells at various time points after IAV/WSN infection. (C) Relative activity of caspase-1 in C24 and L1-3 cells at various time points after IAV/WSN infection against the viability of C24 cells at the start of infection. (**D**) Relative cell viability of C24 and L1-3 cells treated with control IgG and various amounts of anti-IL-1β antibody at various time points after IAV/WSN infection against the viability of each type of cells at the start of infection. (**E**) Western blotting of L1-3 cells treated with 500 ng/mL of control IgG and anti-IL-1β antibody at various time points after IAV/WSN infection for MLKL, phosphorylated MLKL (p-MLKL), and an internal control β-actin. (**F**) IL-1β in the medium of C24 and L1-3 cells transfected with siRNAs for NLRP1b and NLRP3 at various time points after IAV/WSN infection. (**G**) Relative cell viability of C24 and L1-3 cells transfected with siRNAs for NLRP1b and NLRP3 at various time points after IAV/WSN infection against the viability of each type of cells at the start of infection. hpi, hours post infection. Data are the mean ± SD of three independent experiments. *, *P* < 0.05; **, *P* < 0.01; ***, *P* < 0.005; ****, *P* < 0.001.

To investigate the protective mechanism against IAV/WSN-induced necroptosis in prion-infected cells, we assessed the role of inflammasomes in the protection in N2aC24L1-3 cells. IL-1β was abundantly released in the medium of N2aC24L1-3 cells, but not in N2aC24 cells, after IAV/WSN infection (Fig. 3A). Levels of the cleaved active form of caspase-1 and the caspase-1 activity were also higher in N2aC24L1-3 cells than in N2aC24 cells after IAV/WSN infection (Fig. 3B and C). In addition, neutralizing antibody to IL-1β reduced the cell viability of N2aC24L1-3 cells in a dose-dependent manner (Fig. 3D) and increased phosphorylated MLKL in N2aC24L1-3 cells after IAV/WSN infection (Fig. 3E). Moreover, siRNA-mediated knockdown of NLRP3, not NLRP1b, reduced IL-1β release and cell viability in N2aC24L1-3 cells after IAV/WSN infection (Fig. 3F and G). These results suggest that prion-infected cells activate the NLRP3 inflammasome after IAV/WSN infection, thereby suppressing IAV/WSN-induced necroptosis.

### Prion-infected cells highly produce mtROS after IAV/WSN infection

**Fig 4:**
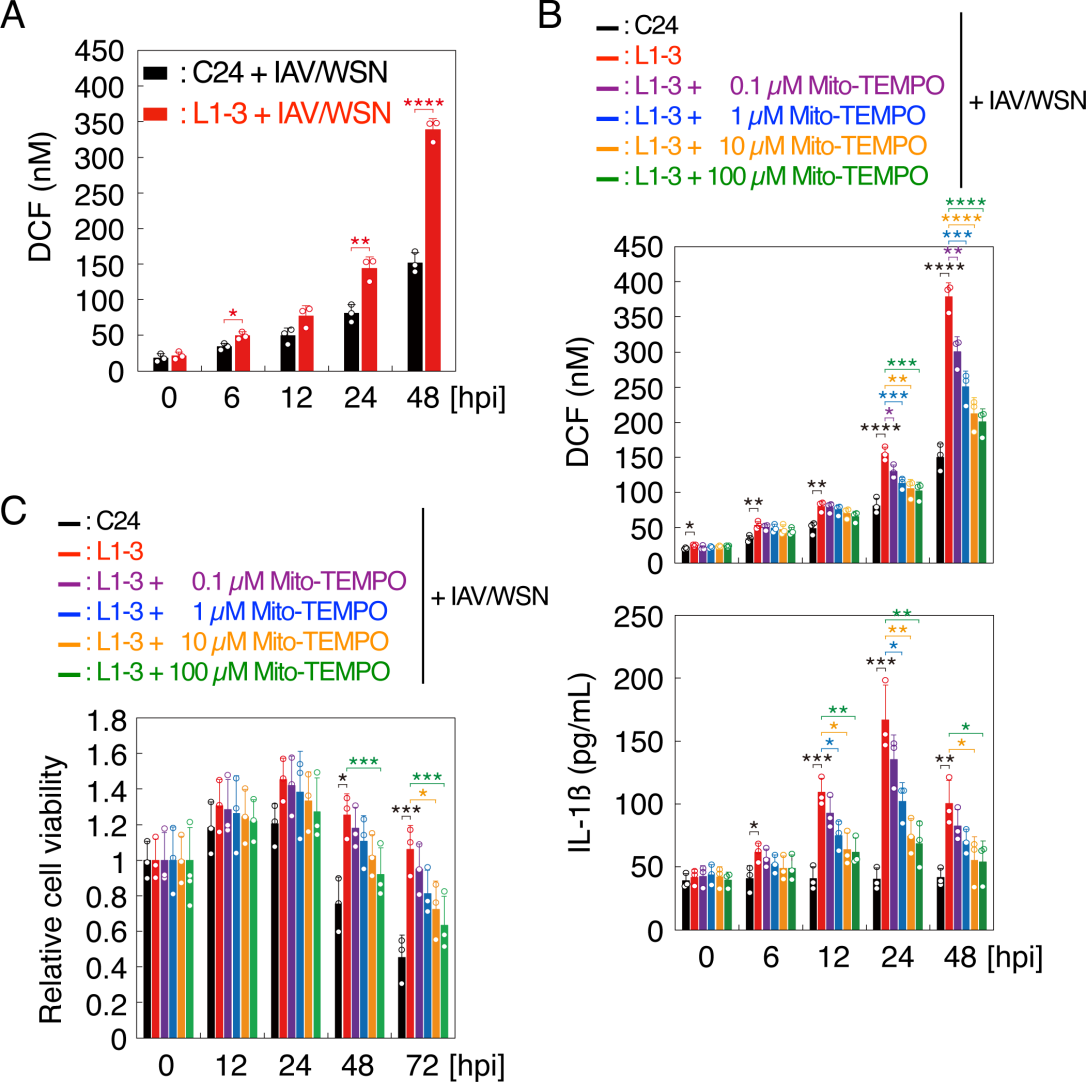
MtROS is highly produced in prion-infected cells after IAV/WSN infection. (**A**) Dichlorofluorescein (DCF) levels representing ROS levels in N2aC24 (**C24**) and N2aC24L1-3 (L1-3) cells at various time points after IAV/WSN infection. (**B**) DCF levels representing ROS levels (upper panel) and IL-1β in the medium (lower panel) in C24 and L1-3 cells treated with various amounts of Mito-TEMPO at various time points after IAV/WSN infection. (**C**) Relative cell viability of C24 and L1-3 cells treated with various amounts of Mito-TEMPO at various time points after IAV/WSN infection against the viability of each type of cells at the start of infection. hpi, hours post infection. Data are the mean ± SD of three independent experiments. *, *P* < 0.05; **, *P* < 0.01; ***, *P* < 0.005; ****, *P* < 0.001.

mtROS is known to activate the NLRP3 inflammasome (32). Therefore, to elucidate the mechanism of NLRP3 inflammasome activation in prion-infected cells after IAV/WSN infection, we measured ROS levels in N2aC24 and N2aC24L1-3 cells after IAV/WSN infection. ROS levels were increased in both cells after IAV/WSN infection (Fig. 4A). However, N2aC24L1-3 cells produced higher levels of ROS than N2aC24 cells after IAV/WSN infection (Fig. 4A). We then treated N2aC24L1-3 cells with Mito-TEMPO, an mtROS-targeting antioxidant, in IAV/WSN infection. Mito-TEMPO not only suppressed ROS levels but also reduced both IL-1β release and cell viability in N2aC24L1-3 cells after IAV/WSN infection in a dose-dependent manner (Fig. 4B and C). Similar results were obtained with N2aC24Chm cells (Fig. S2A through C). These results suggest that prion-infected cells increase mtROS production after IAV/WSN infection, thereby activating the NLRP3 inflammasome.

### *Sod2* gene expression is suppressed in prion-infected cells after IAV/WSN infection

**Fig 5:**
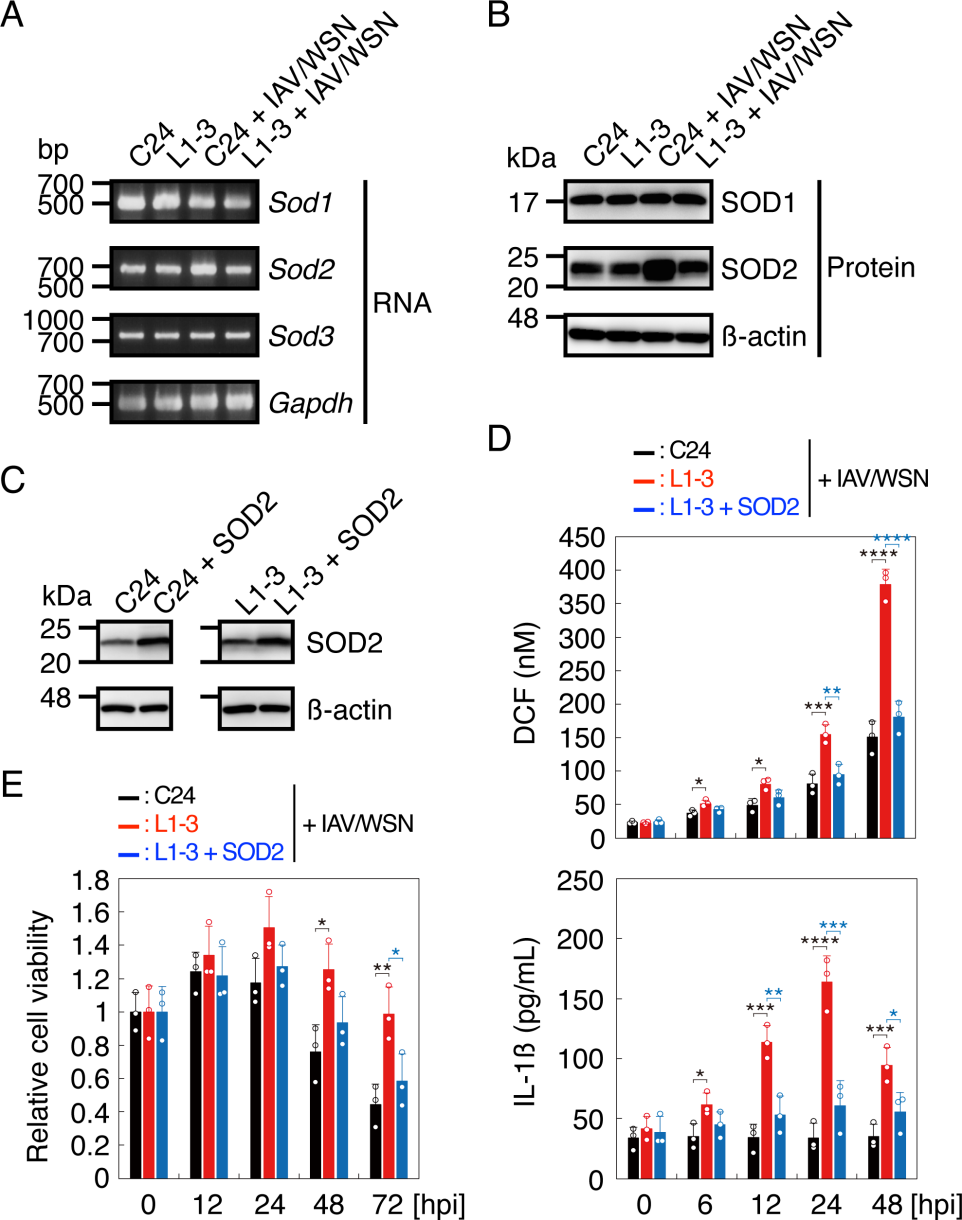
*Sod2* gene expression is suppressed in prion-infected cells after IAV/WSN infection. (**A**) RT-PCR for *Sod1*, *Sod2*, and *Sod3* in N2aC24 (**C24**) and N2aC24L1-3 (L1-3) cells at 48 h after IAV/WSN infection. Glyceraldehyde-3-phosphate dehydrogenase (*Gapdh*) is an internal control. (**B**) Western blotting for SOD1 and SOD2 in C24 and L1-3 cells at 48 h after IAV/WSN infection. β-actin is an internal control. (**C**) Western blotting for SOD2 in C24 and L1-3 cells transfected with pcDNA-SOD2 and control plasmids at 48 h. β-action is an internal control. (**D**) DCF levels representing ROS levels (upper panel) and IL-1β in the medium (lower panel) in C24 and L1-3 cells transfected with control plasmid and L1-3 cells transfected with pcDNA-SOD2 plasmid at various time points after IAV/WSN infection. (**E**) Relative cell viability of C24 and L1-3 cells transfected with control plasmid and L1-3 cells transfected with pcDNA-SOD2 plasmid at various time points after IAV/WSN infection against the viability of each type of cells at the start of infection. hpi, hours post infection. Data are the mean ± SD of three independent experiments. *, *P* < 0.05; **, *P* < 0.01; ***, *P* < 0.005; ****, *P* < 0.001.

To gain insights into the mechanism of the mtROS elevation in prion-infected cells after IAV/WSN infection, we investigated the gene expression of ROS-inactivating SODs, including cytosolic SOD1, mitochondrial SOD2, and extracellular SOD3, in N2aC24 and N2aC24L1-3 cells after IAV/WSN infection. *Sod1* and *Sod3* mRNAs were similarly expressed between IAV/WSN-infected N2aC24 and N2aC24L1-3 cells (Fig. 5A). However, *Sod2* mRNA was increased in N2aC24 cells, but not in N2aC24L1-3 cells, after IAV/WSN infection (Fig. 5A). Consistently, SOD2 protein levels were higher in N2aC24 cells than in N2aC24L1-3 cells after IAV/WSN infection (Fig. 5B). Overexpression of the *Sod2* gene reduced ROS levels, IL-1β release, and cell viability in N2aC24L1-3 cells after IAV/WSN infection (Fig. 5C through E). Similar results were obtained with N2aC24Chm cells (Fig. S3A through E). These results suggest that *Sod2* gene expression is specifically suppressed in prion-infected cells after IAV/WSN infection, therefore increasing ROS levels.

### NF-κB nuclear translocation is disturbed in prion-infected cells after IAV/WSN infection

**Fig 6:**
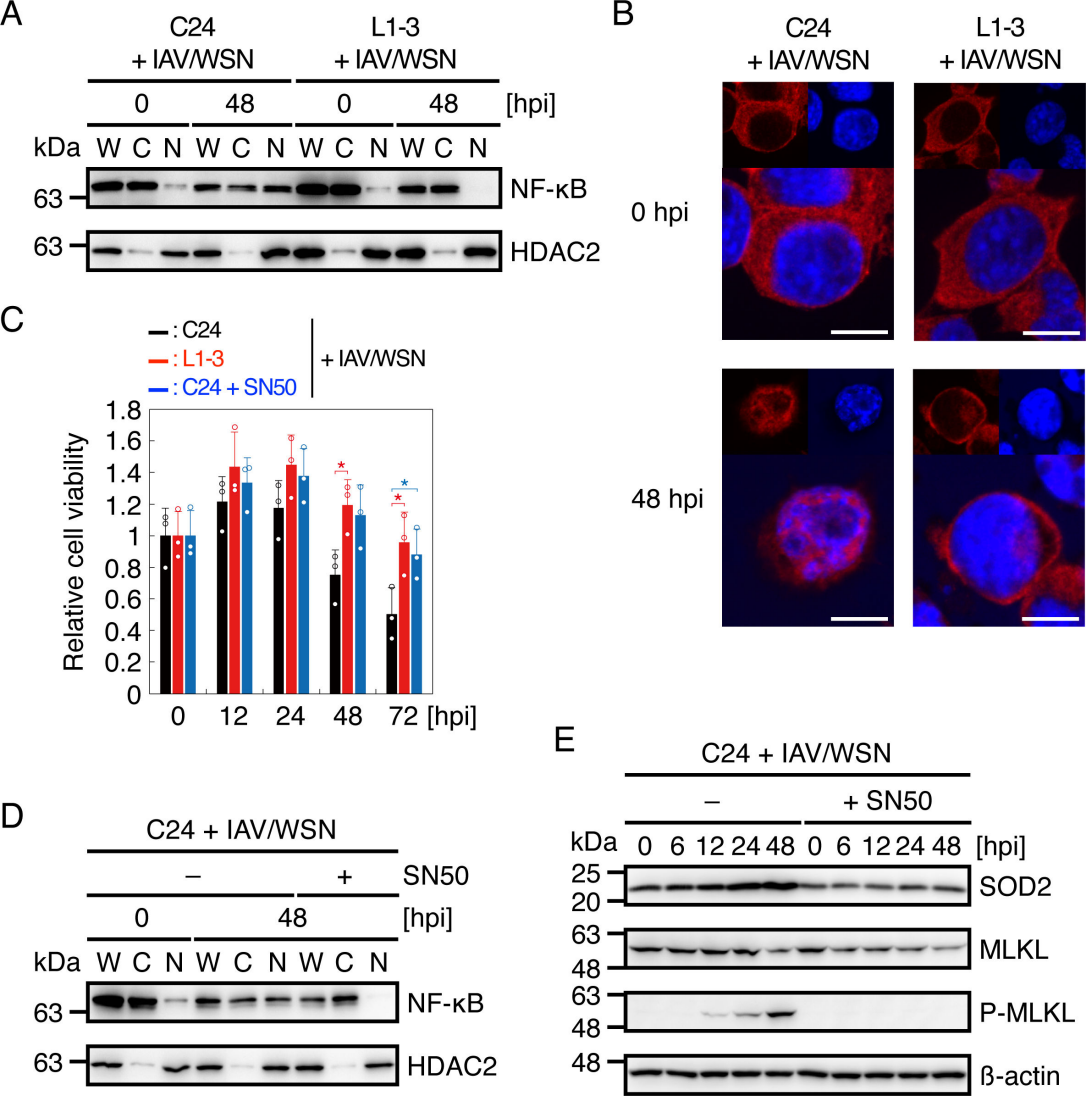
NF-κB nuclear translocation is disturbed in prion-infected cells after IAV/WSN infection. (**A**) Western blotting for the NF-κB p65 in cytoplasmic and nuclear extracts from N2aC24 (**C24**) and N2aC24L1-3 (L1-3) cells at 0 and 48 h after IAV/WSN infection. HDAC2 is a nuclear marker. (**B**) Immunofluorescent staining for NF-κB (red) of C24 and L1-3 cells at 0 and 48 h after IAV/WSN infection. Blue, DAPI; bar 10 µm. (**C**) Relative cell viability of C24 and L1-3 cells and C24 cells treated with 50 µg/mL SN50 at various time points after IAV/WSN infection against the viability of each type of cells at the start of infection. (**D**) Western blotting for NF-κB in cytoplasmic and nuclear extracts from C24 cells treated with and without 50 µg/mL SN50 at 0 and 48 h after IAV/WSN infection. HDAC2 is a nuclear marker. (**E**) Western blotting of C24 cells treated with and without 50 µg/mL SN50 at various time points after IAV/WSN infection for MLKL, phosphorylated MLKL (p-MLKL), and an internal controlβ-actin. W, whole cell lysate; C, cytoplasmic extract; N nuclear extract. hpi, hours post infection. *, *P* < 0.05.

NF-κB is a major transcriptional factor for the *Sod2* gene (33). Therefore, to elucidate the mechanism of the suppressed *Sod2* gene expression in prion-infected cells after IAV/WSN infection, we investigated NF-κB nuclear translocation in N2aC24 and N2aC24L1-3 cells after IAV/WSN infection. Western blotting showed that the NF-κB subunit p65 was less accumulated in the nuclear fraction of N2aC24L1-3 cells than in N2aC24 cells after IAV/WSN infection (Fig. 6A). Immunohistochemistry also showed p65 accumulated in the nucleus of N2aC24 cells, but not in N2aC24L1-3 cells, after IAV/WSN infection (Fig. 6B). Similar results were obtained with N2aC24Chm cells (Fig. S4A and B). Furthermore, the NF-κB nuclear translocation inhibitor SN50 increased cell viability (Fig. 6C). In contrast, SN50 reduced p65 nuclear translocation, SOD2 expression, and phosphorylated MLKL in N2aC24 cells after IAV/WSN infection (Fig. 6D and E). These results indicate that NF-κB nuclear translocation is disturbed in prion-infected cells after IAV/WSN infection, thereby suppressing the *Sod2* gene expression and consequently necroptosis.

### Nuclear translocation signal of cytosolic PrP disturbs NF-κB nuclear translocation in prion-infected cells after IAV/WSN infection

**Fig 7:**
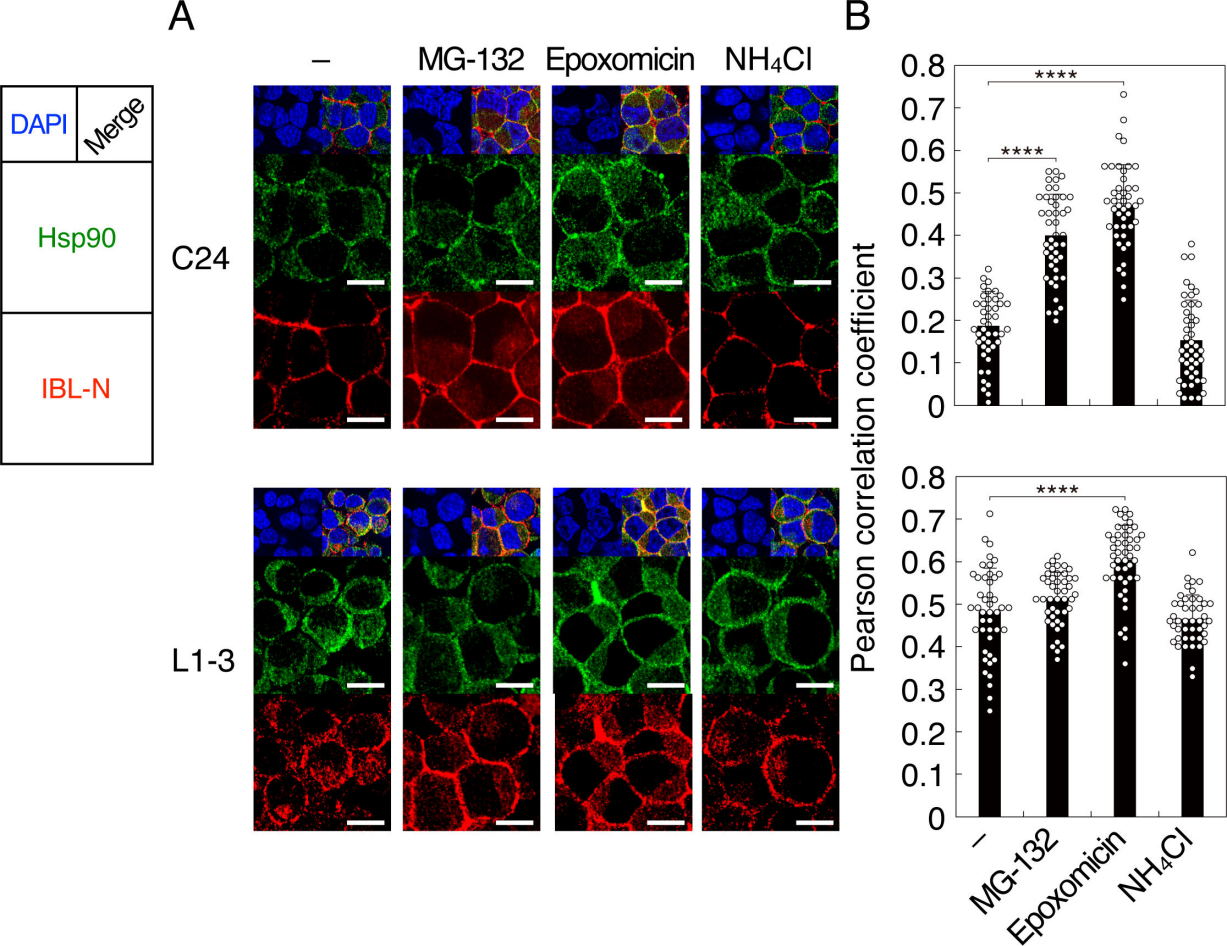
PrP accumulates in the cytoplasm of prion-infected cells. (**A**) Double immunofluorescent staining for Hsp90 (green) and PrP (red) using anti-HSP90 and IBL-N anti-PrP antibodies of N2aC24 (**C24**) (upper panels) and N2aC24L1-3 (L1-3) (lower panels) cells treated with and without 10 µM MG-132, 1 µM epoxomicin, and 10 mM ammonium chloride. Blue, DAPI; bar 10 µm. (**B**) Pearson correlation coefficient for intracellular colocalization of Hsp90 and PrP in C24 (upper panel) cells untreated (*n* = 46) and treated with MG-132 (*n* = 45), epoxomicin (*n* = 45), and ammonium chloride (*n* = 46) and in L1-3 (lower panel) cells untreated (*n* = 44) and treated with MG-132 (*n* = 44), epoxomicin (*n* = 47), and ammonium chloride (*n* = 44). ****, *P* < 0.001.

It has been shown that a portion of PrP molecules is detectable in the cytosol of prion-infected cells and that PrP has a potential nuclear translocation signal in the N-terminus (34, 35). We thus hypothesized that the cytosolic PrP might compete with NF-κB for nuclear translocation through the nuclear translocation signal in prion-infected cells. To explore this idea, we first investigated if cytosolic PrP with the N-terminus could be detectable in prion-infected cells by performing immunohistochemistry of N2aC24 and N2aC24L1-3 cells by using IBL-N anti-PrP antibodies, which recognize the N-terminal residues, after treatment with or without the proteasome inhibitors, MG-132 and epoxomicin, and the lysosome inhibitor NH_4_Cl. Consistent with PrP^C^ being a GPI-anchored membrane glycoprotein, the cell surface was predominantly stained in N2aC24 cells (Fig. 7A). However, in N2aC24L1-3 cells, intracellular staining was evident, whereas cell surface staining was reduced (Fig. 7A). MG-132 and epoxomicin, but not NH_4_Cl, increased intracellular staining for PrP in N2aC24L1-3 cells compared with N2aC24 cells (Fig. 7A). Furthermore, a portion of the intracellular signals of PrP in N2aC24L1-3 cells were colocalized with the cytosolic molecule heat-shock protein 90 (Hsp90) (Fig. 7B). Similar results were obtained with N2aC24Chm cells (Fig. S5). These results suggest that, although PrP molecules are dominantly detectable along the biosynthesis pathway, such as of the cell surface and endosomal compartments, PrP molecules with the N-terminal residues could also be present in the cytosol of prion-infected cells.

**Fig 8:**
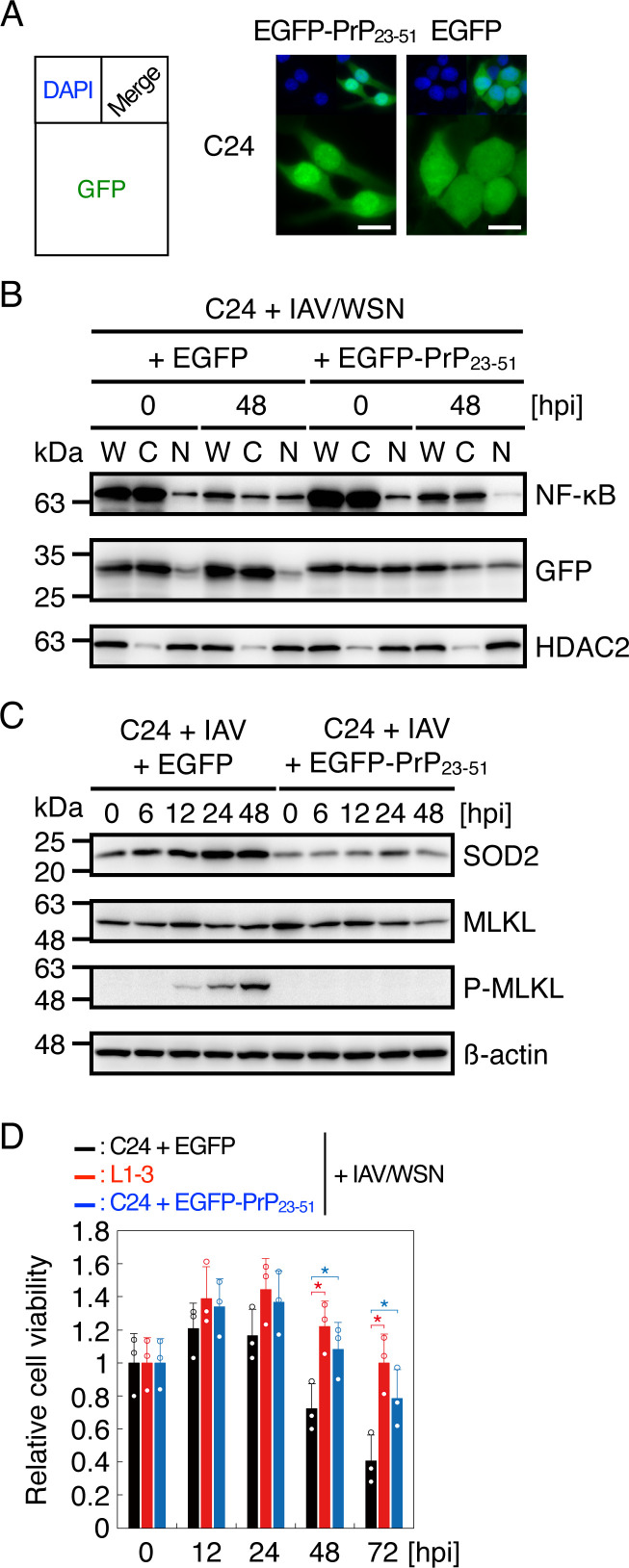
The N-terminal residues 23–51 of PrP function as a potential nuclear translocation signal and disturb NF-κB nuclear translocation after IAV/WSN infection. (**A**) Immunofluorescence analysis for GFP signal (green) in N2aC24 (**C24**) cells transfected with pEGFP-PrP_23-51_ and control plasmids after 48 h. Blue, DAPI; bar 10 µm. (**B**) Western blotting for NF-κB p65 and GFP in cytoplasmic and nuclear extracts from C24 cells transfected with pEGFP-PrP_23-51_ and control plasmids at 0 and 48 h after IAV/WSN infection. HDAC2 is a nuclear marker. W, whole cell lysate; C, cytoplasmic extract; N nuclear extract. (**C**) Western blotting of C24 cells transfected with pEGFP-PrP_23-51_ and control plasmids at various time points after IAV/WSN infection for SOD2, MLKL, phosphorylated MLKL (p-MLKL), and an internal control β-actin. (**D**) Relative cell viability of C24 cells transfected with the pEGFP-PrP_23-51_ and control plasmids and of N2aC24L1-3 (L1-3) cells at various time points after IAV/WSN infection against the viability of each type of cells at the start of infection. hpi, hours post infection. *, *P* < 0.05

The cNLS Mapper predicts the N-terminal residues 23–51 of PrP as a potential nuclear translocation signal. We then transduced the N-terminal residues 23–51 of PrP fused with enhanced green fluorescent protein (EGFP), termed EGFP-PrP_23-51_, into N2aC24 cells and infected them with IAV/WSN. EGFP-PrP_23-51_ was observed in both the cytosol and nucleus, with higher signals in the nucleus (Fig. 8A), confirming that the residues 23–51 potentially function as a nuclear translocation signal. In EGFP-PrP_23-51_-transduced N2aC24 cells, p65 nuclear translocation was disturbed (Fig. 8B), SOD2 expression and phosphorylated MLKL were decreased (Fig. 8C), and cell viability was increased after IAV/WSN infection (Fig. 8D). Taken together, these results support the idea suggesting that cytosolic PrP accumulated in prion-infected cells might disturb NF-κB nuclear translocation through the nuclear translocation signal after IAV/WSN infection, thereby decreasing SOD2 expression and phosphorylated MLKL and consequently increasing cell viability in prion-infected cells.

## DISCUSSION

In this study, we showed that, compared with prion-uninfected control N2aC24 cells, two different prion-infected cells, N2aC24L1-3 and N2aC24Chm cells, activated the NLRP3 inflammasome after IAV/WSN infection and were highly resistant to IAV/WSN-induced necroptosis, suggesting that PrP^Sc^-prions might play a functional role in activation of the NLRP3 inflammasome and suppression of necroptosis after viral infections.

We showed that IAV/WSN infection increased the levels of the apoptosis marker cleaved caspase-3, the autophagic cell death marker LC3-II, and the necroptosis marker phosphorylated MLKL in prion-uninfected cells. These results are consistent with the fact that IAV infection could activate multiple cell death pathways *in vitro* and *in vivo* (36). However, phosphorylation of MLKL were markedly prevented in prion-infected cells after IAV/WSN infection, although other cell death markers were similarly increased between prion-uninfected and prion-infected cells. These results suggest that necroptosis, but not other types of cell death, is prevented in prion-infected cells after IAV/WSN infection, therefore prion-infected cells exhibiting partial resistance to IAV/WSN-induced cell death.

We found that NF-κB nuclear translocation was disturbed in prion-infected cells after IAV/WSN infection, resulting in suppression of the *Sod2* gene expression and elevation of mtROS levels, and that the elevated mtROS in turn activated NLRP3 inflammasomes and eventually suppressed necroptotic cell death in prion-infected cells after IAV/WSN infection. These results suggest that PrP^Sc^-prions might disturb NF-κB nuclear translocation after IAV/WSN infection, thereby stimulating the NLRP3 inflammasome and suppressing necroptosis. We also showed that EGFP-PrP_23-51_ disturbed the nuclear translocation of the NF-κB subunit p65 in N2aC24 cells after IAV/WSN infection. Residues 23–51 include a potential nuclear translocation signal (35). Indeed, EGFP-PrP_23-51_ was predominantly detected in the nucleus of N2aC24 cells. It is thus possible that PrP_23-51_ might interfere with the nuclear translocation of p65 through the nuclear translocation signal. Immunofluorescent cell staining with IBL-N antibodies against the PrP N-terminal residues exhibited strong cell surface staining and weak intracellular staining in prion-infected cells. A portion of the intracellular PrP signals were partly colocalized with the cytosolic marker Hsp90 and increased after treatment with a proteasome inhibitor. These results suggest that, although PrP molecules are dominantly present on the cell surface and in endosomal compartments, a proportion of PrP molecules with the N-terminal residues could be localized in the cytosol of prion-infected cells. Other investigators also detected PrP in the cytosol of prion-infected cells (34, 37, 38). p65 needs to bind to nuclear transporter molecules, such as importins through the nuclear translocation signal for its nuclear transport (39). It is therefore conceivable that cytosolic PrP in prion-infected cells might compete with p65 for nuclear transporter molecules, such as importins, through the nuclear translocation signal located in residues 23–51 after IAV/WSN infection.

The exact molecular nature of cytosolic PrP in prion-infected cells remains to be determined. PrP^C^ is a GPI-anchored membrane glycoprotein, predominantly localized on the cell surface and to lesser extents in the biosynthetic and endocytic membranous compartments (38). However, cytosolic PrP has been reported even in prion-uninfected culture cells and subpopulations of neurons in normal mice (37, 40, 41). Consistent with these reports, by immunofluorescent cell staining of N2aC24 cells, we detected weak but distinctive cytosolic PrP signals, which were colocalized with the cytosolic marker Hsp90 and increased after treatment with proteasome inhibitors. It has been postulated that a portion of PrP molecules retrogradely translocates from the endoplasmic reticulum (ER) into the cytosol due to their improper folding in the ER or fails to enter the ER from the cytosol due to the insufficient signal sequence of PrP (42). PrP^Sc^ has been shown to cause ER stress, which is known to increase the retrograde translocation of proteins from the ER to the cytosol (40, 41, 43, 44). It is thus possible that PrP^Sc^ might increase cytosolic PrP levels in prion-infected cells by causing ER stress. The transmembrane form of PrP, termed ^Ctm^PrP, which exposes the N-terminus in the cytosol and the C-terminus in the extracellular space, has been also reported to be increased in prion-infected mouse brains (45). Moreover, using immunostaining after formic acid pretreatment to remove PrP^C^, a portion of PrP^Sc^ was shown to colocalize with the cytosolic marker Hsc70 in RML-infected ScN2aPK-1 cells (34), suggesting that PrP^Sc^ itself could also be translocated into the cytosol in prion-infected cells. Identification of the exact molecular nature of cytosolic PrP would be worthy of further clarification of the mechanism underlying the cytosolic accumulation of PrP in prion-infected cells.

Cytosolic PrP or ^Ctm^PrP has been suggested to play a pathogenic role in prion diseases (41, 46, 47). Indeed, transgenic mice for cytosolic PrP, which consists of residues 23–230, or ^Ctm^PrP-favoring PrP mutants have shown to spontaneously develop prion disease-like neurodegeneration (41, 46). Chakrabarti and Hegde reported that cytosolic PrP and ^Ctm^PrP could interact and functionally interfere with the cytosolic ubiquitin ligase Mahogunin (47), whose functional loss causes spongiform neurodegeneration in animals. Kristiansen et al. also reported that the proteolytic activity of the 26S proteasome was impaired in RML-infected cells and 22L-infected mouse brains due to interaction with PrP^Sc^ (34). Thus, cytosolic PrP or cytosolic PrP^Sc^ or ^Ctm^PrP may play a pathogenic role in prion diseases by functionally disturbing cellular molecules, including the 26S proteasome and Mahogunin. We showed that cytosolic PrP could functionally disturb NF-κB in prion-infected cells after IAV/WSN infection by inhibiting the nuclear translocation of p65. However, mice deficient for p65 were shown to develop prion disease similarly to control wild-type mice after intracerebral inoculation with RML6 prions (48), suggesting the unlikelihood that the cytosolic PrP-mediated disturbance of the NF-κB function is relevant to prion pathogenesis. Yet, it remains possible that cytosolic PrP might disturb the nuclear translocation of other molecule(s) crucial for prion pathogenesis. Identification of such a molecule(s) would contribute to understanding of the pathogenic mechanism of prion diseases.

Causal relationships have been suggested between viral infections and other types of protein misfolding-associated neurodegenerative disorders, including Alzheimer’s disease (AD), Parkinson’s disease (PD), and amyotrophic lateral sclerosis (ALS) (49, 50). Herpes simplex virus type-1 (HSV-1) is suggested to etiologically link to AD (51), IAV and Japanese encephalitis virus to PD (52, 53), and enteroviruses and human herpesviruses to ALS (54, 55). We previously showed that PrP^C^ was converted into infectious PrP^Sc^-prions in N2aC24 cells after IAV/WSN infection (56), suggesting that IAV infection in neurons might be a causative event for sporadic prion diseases. Interestingly, amyloid-β (Aβ) aggregates were shown to play a protective role against infection with HSV-1 and other human herpesviruses by binding to and thereby entrapping the viruses in an AD mouse model and 3-dimensional human neuronal cell culture (57). We showed here that prion-infected cells were higher in resistance to IAV/WSN infection than control prion-uninfected cells, by increasing cytosolic PrP and subsequently activating the NLRP3 inflammasome and suppressing necroptosis, suggesting the protective role for PrP^Sc^ prions against IAV/WSN infection. The fungus [Het-s] prion was shown to activate the so-called heterokaryon incompatibility and thereby play a protective role against the spread of mycoviruses and other parasites between incompatible strains (22, 23), although it remains controversial whether or not yeast prions, such as [*URE3*], [*PSI^+^*], and [*MOD^+^*], have a cellular protective role against environmental stress (10, 13–16). It is thus conceivable that not only PrP^Sc^-prions but also other prion-like protein aggregates in neurodegenerative disorders might be produced as a protective cellular mechanism against various environmental stress, including viral infections. Further elucidation of the exact protective mechanism of PrP^Sc^-prions against IAV/WSN infection would be helpful for understanding the protective mechanism of other prion-like protein aggregates against environmental stress.

## MATERIALS AND METHODS

### Antibodies (Abs)

Abs used in this study are 6D11 mouse anti-PrP Ab (808003; BioLegend, San Diego, CA), SAF83 mouse anti-PrP Ab (A03207; Bertin Bioregent, Montigny le Bretonneux, France), rabbit IBL-N anti-PrP Abs (18635; Immuno-Biological Laboratories, Gunma, Japan), anti-influenza-HA Abs (GTX127357; GeneTex, Irvine, CA), anti-influenza-NP Abs (GTX125989; GeneTex), anti-influenza-M2 Abs (GTX125951; GeneTex), mouse anti-ß-actin Ab (M177-3; MBL Life Science, Tokyo, Japan), rabbit anti-caspase-3 Abs (9662; Cell Signaling Technology, Danvers, MA), rabbit anti-cleaved-caspase-3 Ab (9664, Cell Signaling Technology), rabbit anti-LC3B Ab (3868; Cell Signaling Technology), rabbit anti-MLKL Ab (37705; Cell Signaling Technology), mouse anti-phosphorylated-MLKL (Ser345) Ab (MABC1158; Millipore, Burlington MA), mouse anti-RIP3 Ab (sc-374639; Santa Cruz Biothechnology, Dallas, TX), rabbit anti-phosphorylated-RIP3 (Thr231, Ser232) Ab (ab222320; abcam, Cambridge, United Kingdom), rabbit anti-caspase-1 Ab (NBP1-96553; Novus Biologicals, Centennial, CO), rabbit anti-SOD1 Abs (Ab13498; abcam), rabbit anti-SOD2 Abs (ab13533, abcam), rabbit anti-NF-κB Ab (8242; Cell Signaling Technology), mouse anti-HDAC2 Ab (5113; Cell Signaling Technology), rabbit anti-GFP Abs (A1122; Invitrogen, Waltham, MA), mouse anti-HSP90 Ab (60318–1-lg; Proteintech, Rosemont, IL), anti-mouse IgG horseradish peroxidase (HRP)-linked Ab (NA931; GE Healthcare, Little Chalfont, England), anti-rabbit IgG HRP-linked Ab (NA934; GE Healthcare), Alexa Fluor 488-conjugated goat anti-mouse IgG Abs (A11001; Thermo Fisher Scientific, Rockford, IL), and Alexa Fluor 594-conjugated goat anti-rabbit IgG Abs (A11012; Thermo Fisher Scientific).

### Plasmid construction

A mouse *Sod2* cDNA fragment was amplified from mouse brain QUICK-clone cDNA (637301; Clontech Laboratories, Mountain View, CA) by using PCR (primers; 5’-gggggatccatgttgtgtcgggcggcgtg-3’, 5’-gggctcgagtcacttcttgcaagctgtgt-3’), and the amplified fragment was subcloned into pCR2.1-TOPO vector (451641; Invtrogen). After DNA sequence confirmation, it was inserted into *Bam*H I/*Xho* I-digested pcDNA3.1(+) (V79020; Invitrogen), resulting in pcDNA-SOD2. The plasmid pEX-A2J2-PrP_23-51_, which has a synthetic DNA fragment encoding the N-terminal residues 23–51 of PrP with a *Bgl* II site at the 5′ end and a stop codon (TGA) and a *Sal* I site at the 3′ end, was purchased from Eurofin Genomics (Ebersberg, Germany). A PrP_23-51_ fragment was then ligated into *Bgl* II/*Sal* I digested pEGFP-C1 to obtain pEGFP-PrP_23-51_.

### Cells

N2aC24, N2aC24L1-3, N2aC24Chm, cured N2aC24L1-3, and cured N2aC24Chm cells (29, 31) were maintained at 37°C with 5% CO_2_ in air in Dulbecco’s Modified Eagle Medium (D-MEM) (043–30085; Wako Pure Chemical Industries, Osaka, Japan) supplemented with 1× Penicillin-Streptomycin Mixed Solution (26253–84; Nakalai Tesque, Osaka, Japan), 100 U/mL penicillin, 100 µg/mL streptomycin, and 10% heat-inactivated fetal bovine serum (FBS) (10437–028; Gibco, Rockford, IL). Cells were split at 10- to 15-fold dilution every 4 to 5 days.

### Preparation of virus, determination of virus titer, and viral infection of cells

Preparation of IAV/WSN and determination of its titer were performed as described previously (58). Viral infection of cells was also performed as described previously (56). In brief, cells were cultured in six-well plates at a density of 6 × 10^5^ cells/well for 16 h, and then infected with 1.0 multiplicity of infection (MOI) of IAV/WSN. Living cells were counted using Trypan Blue staining (0.4%; 207–03252; Wako Pure Chemical Industries) and cell viability was indicated as the number of living cells.

### Treatment with anti-IL-1β Abs

Goat anti-IL-1β Abs (AF-401-NA; R&D Systems, Minneapolis, MN) and mouse polyclonal IgG (I5381-10MG; Sigma-Aldrich, St. Louis, MO) were added to cell culture medium at indicated concentrations. The cells were simultaneously infected with IAV/WSN at 1 MOI.

### Treatment with Mito-TEMPO

Mito-TEMPO (sc-221945; Sigma-Aldrich) was added to cell culture medium at indicated concentrations. The cells were simultaneously infected with IAV/WSN at 1 MOI.

### Overexpression of SOD2 and PrP_23-51_

Cells were cultured in a six-well plate at a density of 1 × 10^6^ cells/well and transiently transfected with pcDNA, pcDNA-SOD2, pEGFP-C1, and pEGFP-PrP_23-51_ at the final concentration of 2.4 µg/mL using Lipofectamine 2000 (11668019; Invitrogen) as recommended in the instruction manual. At 6 and 24 h after transfection with pcDNA and pcDNA-SOD2 and with pEGFP-C1 and pEGFP-PrP_23-51_, respectively, cells were washed with PBS and infected with IAV/WSN at 1 MOI.

### Knockdown of NLRP1B and NLRP3

Cells were cultured in a six-well plate at a density of 1 × 10^6^ cells/well. The expression of NLRP1B and NLRP3 was knocked down using MISSION siRNA (Sigma-Aldrich): NLRP3, siRNA ID: SASI_Mm01_00136572; NLRP1B, siRNA ID: SASI_Mm01_00020723; Negative control, siRNA ID: SASI_Mm01_00089196. Each siRNA was transfected into cells at a final concentration of 13 nM using Lipofectamine RNAiMax (13778150; Invitrogen). At 48 h after transfection, cells were washed with PBS and infected with IAV/WSN at 1 MOI.

### Treatment with SN50

SN50 peptide (17493; Cayman Chemical, Ann Arbor, MI), a cell-permeable peptide that blocks the nuclear import of NF-κB, was added to cell culture medium at 50 µg/mL, and the cells were simultaneously infected with IAV/WSN at 1 MOI.

### Treatment of cells with proteasome and lysosome inhibitors

MG-132 (474790; Sigma-Aldrich), a cell permeable proteasome inhibitor, Epoxomicin (AG-CN2-0422; AdipoGen Life Sciences, Füllinsdorf, Switzerland), a cell permeable proteasome inhibitor, and ammonium chloride (02424–55; Nakalai Tesque), lysosome inhibitor, were added to cell culture medium at concentrations of 10 µM, 1 µM, and 10 mM for 16 h, respectively. Immunofluorescence analysis was then performed.

### Preparation of cell lysates and protease K treatment

All procedures were performed at 4°C unless otherwise stated. The cells were washed thrice with PBS (11482–15; Nakalai Tesque), lysed in a lysis buffer (20 mM Tris-HCl, pH 7.4, 0.5% Triton X-100, 0.2% sodium deoxycholate, 100 mM NaCl), and then centrifuged at 1,500×*g* for 5 min to remove insoluble debris. Protein concentration in the lysate was measured using the bicinchoninic acid (BCA) protein assay kit (23225; Pierce, Rockford, IL) with bovine serum albumin (23209; Pierce) as a standard and adjusted to 3 mg of protein/mL with the lysis buffer. Subsequently, 100 µL of the lysates were digested with 6 µg PK (final concentration, 20 µg PK/mg proteins; 165–21043; Wako Pure Chemical Industries) at 37°C for 30 min. The samples were finally mixed with the sodium dodecyl sulfate (SDS) sample buffer (62.5 mM Tris-HCl [pH 6.8], containing 5% SDS, 4% β-mercaptoethanol, 5% glycerol, 0.04% bromophenol blue, 3 mM EDTA) and heated at 95°C for 10 min before being subjected to Western blotting.

### Western blotting

Western blotting was performed as described previously (56). The signals were measured densitometrically using the LAS-4000 mini-chemiluminescence imaging system (Fuji Film, Tokyo, Japan) and Image Gauge software (Fuji Film).

### Lactate dehydrogenase (LDH) assay

LDH assay was performed using the LDH cytotoxicity detection kit (MK401; Takara bio, Shiga, Japan) according to the manufacturer’s protocol. In brief, culture supernatant was collected at indicated times, and the reaction mixture was added to it. After 30-min incubation at room temperature, the absorbances of samples were measured at 492 nm and 630 nm using a plate reader (Varioskan Flash; Thermo Fisher Scientific).

### Enzyme-linked immunosorbent assay (ELISA) for IL-1β

IL-1β levels in culture supernatant were measured using the Quantkine ELISA mouse IL-1ß immunoassay Kit (MLB00C; R&D Systems) according to the manufacturer’s protocol. In brief, collected culture supernatant was diluted 1:1 with the assay diluent supplied in the kit and added to the ELISA microplate wells. After 2 h at room temperature, the wells were washed five times with wash buffer. Mouse IL-1β conjugate was added for 2 h. The wells were then washed with wash buffer, and substrate reagents were added for 30 min. The reaction was stopped by adding the stop solution. The absorbances of the samples at 450 nm were measured using a plate reader (Varioskan Flash). The amount of IL-1β in each culture supernatant was calculated using the standard curve of recombinant IL-1β.

### Measurement of caspase-1 activity

One hundred fifty microliters of 1 mg protein/mL cell lysate and a final concentration of 5 mM dithiothreitol and 250 µM Ac-YVAD-MCA (3161 v; Peptide Institute, Osaka, Japan) were incubated in black 96-well plates at 37°C for 1 h. The fluorescence intensity of 7-amino-4-methylcoumarin (AMC), which is produced by cleavage of MCA by caspase-1, was measured using a plate reader (Varioskan Flash) with an excitation wavelength of 380 nm and an emission wavelength of 460 nm.

### Measurement of ROS levels

ROS levels in the cells were measured using the OxiSelect Intracellular ROS Assay Kit (STA-342; Cell Biolabs, San Diego, CA) according to the manufacturer’s protocol. In brief, the cells were seeded on black 96-well plate. At 1 h before IAV/WSN infection, the cells were washed with PBS, then 100 µL of 1 x DCFH-DA/media solution is added to the cells and incubated at 37°C for 1 h. After reaction with DCFH-DA/medium solution, the cells were washed with PBS and infected with IAV/WSN at 1 MOI. ROS levels in the cells were calculated from fluorescence intensity at an excitation wavelength of 480 nm and an emission wavelength of 530 nm using a plate reader (Varioskan Flash).

### Real time (RT)-PCR

Total RNA was first extracted from cells using RNeasy Mini Kit (74104; QIAGEN, Hilden, Germany) according to the manufacturer’s protocol. Briefly, the cells were homogenized in buffer RLT and transferred to a QIAshredder spin column (QIAGEN). The flow-through was mixed with an equal volume of 70% ethanol and applied to an RNeasy spin column (QIAGEN). Total RNA bound to the column was washed with buffer RW1, then buffer RPE, and eluted with RNase-free water. RT-PCR was performed using the OneStep RT-PCR Kit (210212; QIAGEN) according to the manufacturer’s protocol. Briefly, 8 ng of total RNA was then mixed with primers, dNTPs, and OneStep RT-PCR enzyme mix. The mixture was incubated at 50°C for 30 min and then subjected to PCR reaction (initial PCR activation step at 95°C for 15 min; 3-step cycling: denaturation at 94°C for 30 s, annealing at 60°C for 30 s, extension at 72°C for 1 min; final extension at 72°C for 10 min). For each gene, the primer sequences and the number of PCR cycles are shown in Table S1. The products were analyzed by 1.5% agarose gels.

### Cell fractionation

Cell fractionation was performed using the NE-PER Nuclear and Cytoplasmic Extraction Reagents kit (78833; Thermo Fisher Scientific). In brief, the cells were dissolved in ice-cold CER I solution, then ice-cold CER II and CER III solutions were added. The supernatant (cytoplasmic extract) was separated by centrifugation. The remaining insoluble fraction was suspended in ice-cold NER solution, and the supernatant (nuclear extract) by centrifugation.

### Immunofluorescence analysis

All manipulations were done at room temperature unless otherwise stated. The cells on coverslips (15 mm No. 1; Matsunami Glass Ind., LTD, Osaka, Japan) were washed thrice with PBS and fixed with 4% paraformaldehyde for 15 min. The cells were then washed thrice with PBS and permeabilized with 0.1% Triton X-100 in PBS for 4 min. After washing thrice with PBS, the cells were incubated with 5% FBS in PBS for 30 min for blocking and then with the first antibodies in 0.5% FBS in PBS at 4°C overnight. After removal of the excess antibodies by washing with PBS thrice, the cells were incubated with the secondary antibodies and 4’,6-Diamidino-2-phenylindole (DAPI) (340–07971; Dojindo Laboratories, Kumamoto, Japan) in PBS for 2 h. After washing thrice with PBS, the coverslips were mounted with CC/Mount (K002; Diagnostic Biosystems, Pleasanton, CA). Fluorescence images were visualized using BZ-810 (Keyence, Osaka, Japan) and analyzed with BZ-800 analyzer software (Keyence). To evaluate the co-localization of proteins of interest, Pearson’s correlation coefficient was calculated using Fiji version of ImageJ, a free image processing software (https://fiji.sc).

### cNLS mapper prediction

The NLS sequence was predicted using the cNLS Mapper (http://nls-mapper.iab.keio.ac.jp/cgi-bin/NLS_Mapper_form.cgi) (59). For the cNLS Mapper analysis, the cutoff score was set to 3.0, and the protein region searched for bipartite NLSs with long linkers was within the terminal 60 amino acid region.

### Data analysis and visualization

Data analysis and visualization were performed using Python (version 3.12.1) programming language, utilizing Pandas (version 2.1.4), Matplotlib (version 3.8.2), and Seaborn (version 0.13.1) for data manipulation and generating plots. Statistical analyses were conducted using a Student’s *t*-test (Microsoft EXCEL for Mac software, version 16.80). Figures were prepared using Adobe Illustrator (version 28.1).

## Data Availability

The data sets of this study are available from the corresponding author upon reasonable request. All data are included in this published article and its supplemental material files.

## SUPPLEMENTAL MATERIAL

Supplemental figures and table jvi.01262-24-s0001.pdf Fig. S1 to S5; Table S1.

ASM does not own the copyrights to Supplemental Material that may be linked to, or accessed through, an article. The authors have granted ASM a non-exclusive, world-wide license to publish the Supplemental Material files. Please contact the corresponding author directly for reuse.
