# Supplementary material for: Protective role of cytosolic prion protein against virus infection in prion-infected cells
Source: J Virol. 2024 Aug 28;98(9):e01262-24. doi: 10.1128/jvi.01262-24 (PMC11406989; doi:10.1128/jvi.01262-24)
Supplement: Supplemental figures and table — Fig. S1 to S5; Table S1. [file jvi.01262-24-s0001.pdf]

Supplemental Material

Protective Role of Cytosolic Prion Protein against Virus Infection in Prion-Infected Cells

Hideyuki Hara, Junji Chida, Batzaya Batchuluun, Etsuhisa Takahashi, Hiroshi Kido, Suehiro Sakaguchi

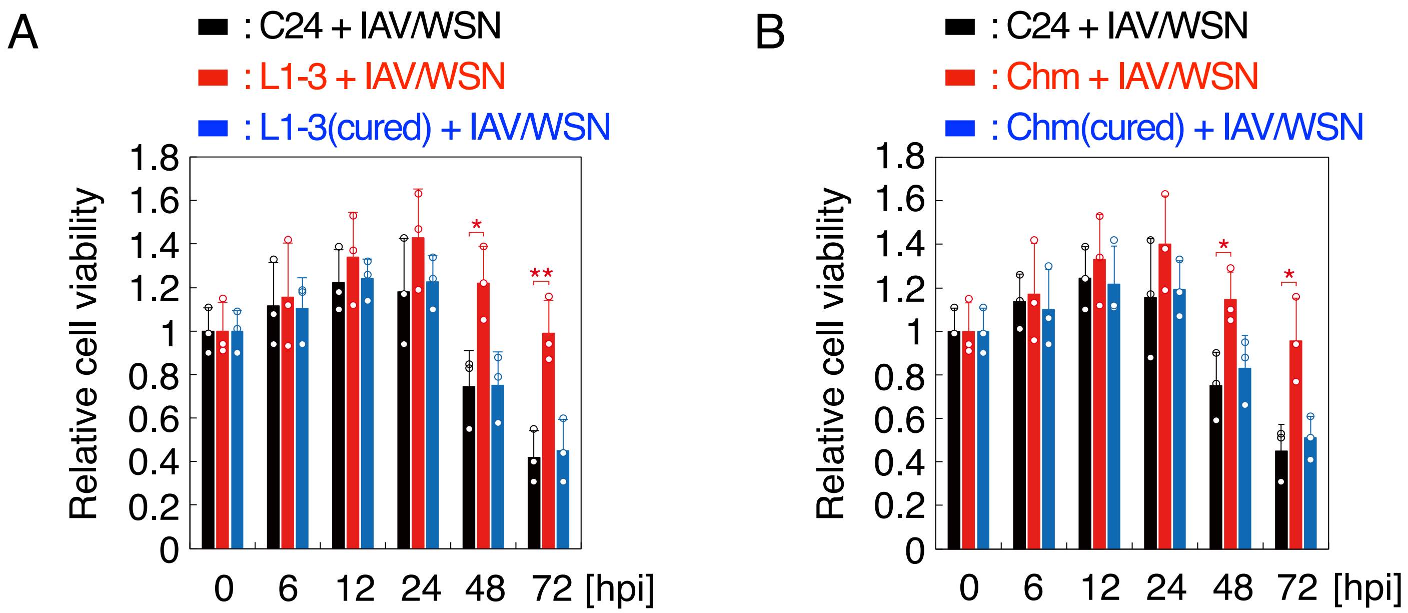

Fig. S1 Prion-infected cells are partially resistant to IAV/WSN infection. **(A)** Relative cell viability of N2aC24 (C24), N2aC24L1-3 (L1-3), and cured L1-3 [L1-3(cured)] cells at various time points after IAV/WSN infection against the viability of each type of cells at the start of infection. **(B)** Relative cell viability of C24, N2aC24Chm (Chm), and cured Chm [Chm(cured)] cells at various time points after IAV/WSN infection against the viability of each type of cells at the start of infection.

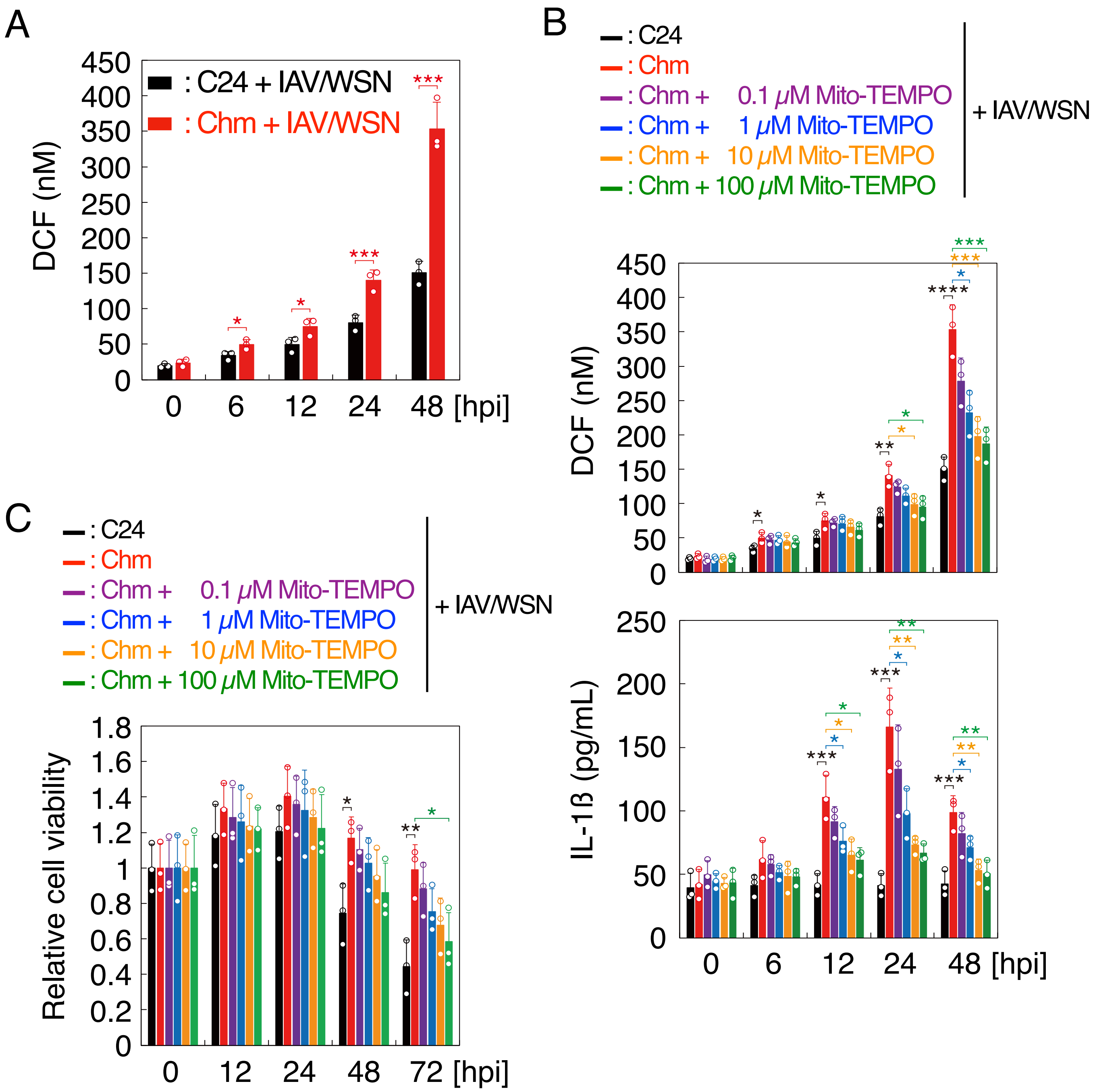

Fig. S2 MtROS is highly produced in prion-infected cells after IAV/WSN infection. **(A)** DCF levels representing ROS levels in N2aC24 (C24) and N2aC24Chm (Chm) cells at various time points after IAV/WSN infection. **(B)** DCF levels representing ROS levels (upper panel) and IL-1b in the medium (lower panel) in C24 and Chm cells treated with various amounts of Mito-TEMPO at various time points after IAV/WSN infection. **(C)** Relative cell viability of C24 and Chm cells treated with various amounts of Mito-TEMPO at various time points after IAV/WSN infection against the viability of each type of cells at the start of infection. hpi, hours post infection. Data are the mean ± SD of three independent experiments. *, $p < 0.05$; **, $p < 0.01$; ***, $p < 0.005$; ****, $p < 0.001$

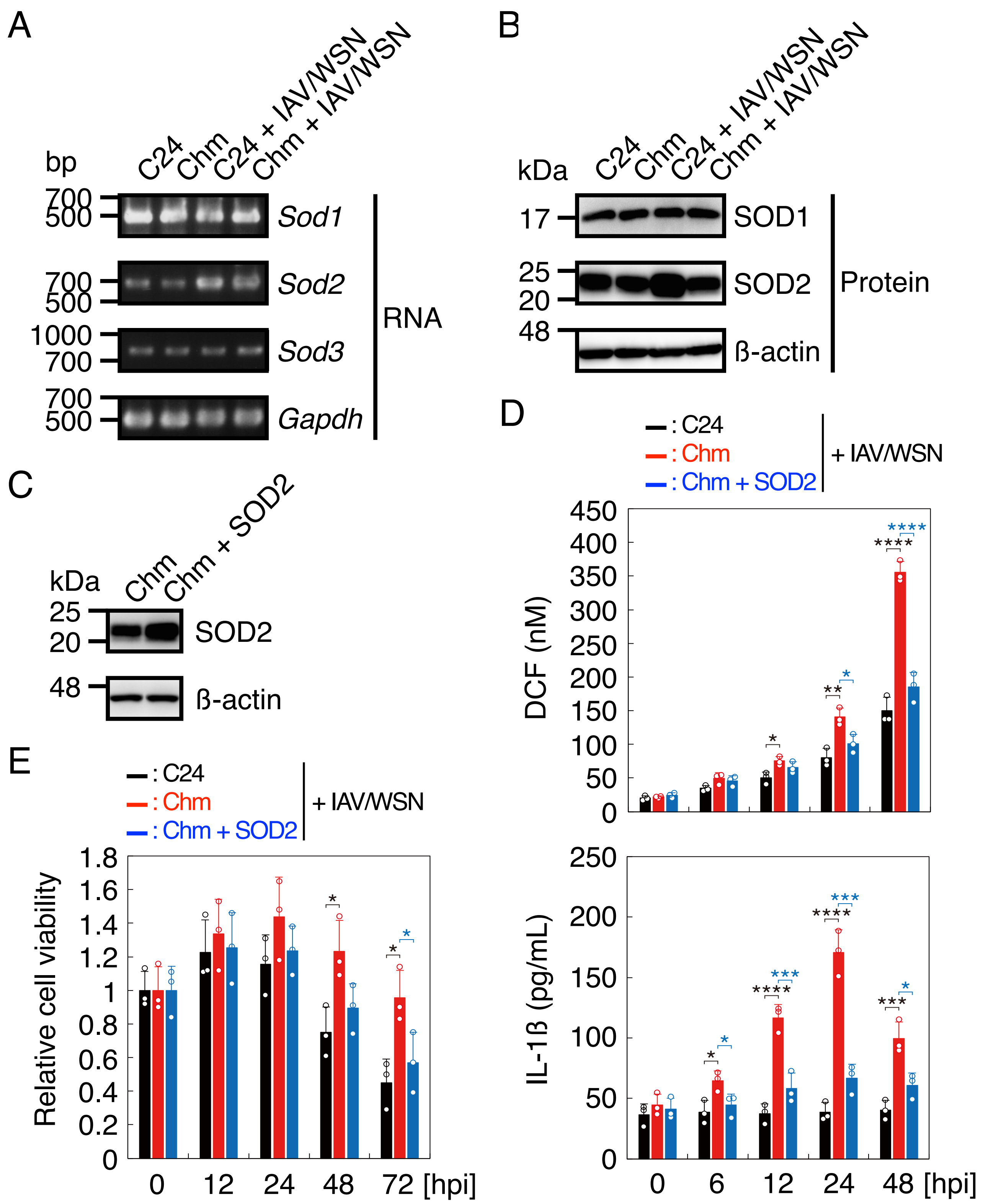

Fig. S3 *SOD2* gene expression is suppressed in prion-infected cells after IAV/WSN infection. **(A)** RT-PCR for *Sod1*, *Sod2*, and *Sod3* in N2aC24 (C24) and N2aC24Chm (Chm) cells at 48 hours after IAV/WSN infection. Glyceraldehyde-3-phosphate dehydrogenase (*Gapdh*) is an internal control. **(B)** Western blotting for SOD1 and SOD2 in C24 and Chm cells at 48 hours after IAV/WSN infection. ß-actin is an internal control. **(C)** Western blotting for SOD2 in C24 and Chm cells transfected with pcDNA-SOD2 and control plasmids at 48 hours. b-action is an internal control. **(D)** DCF levels representing ROS levels (upper panel) and IL-1ß in the medium (lower panel) in C24 and Chm cells transfected with control plasmid and Chm cells transfected with pcDNA-SOD2 plasmid at various time points after IAV/WSN infection. **(E)** Relative cell viability of C24 and Chm cells transfected with control plasmid and Chm cells transfected with pcDNA-SOD2 plasmid at various time points after IAV/WSN infection against the viability of each type of cells at the start of infection. hpi, hours post infection. Data are the mean ± SD of three independent experiments. *, $p < 0.05$; **, $p < 0.01$; ***, $p < 0.005$; ****, $p < 0.001$

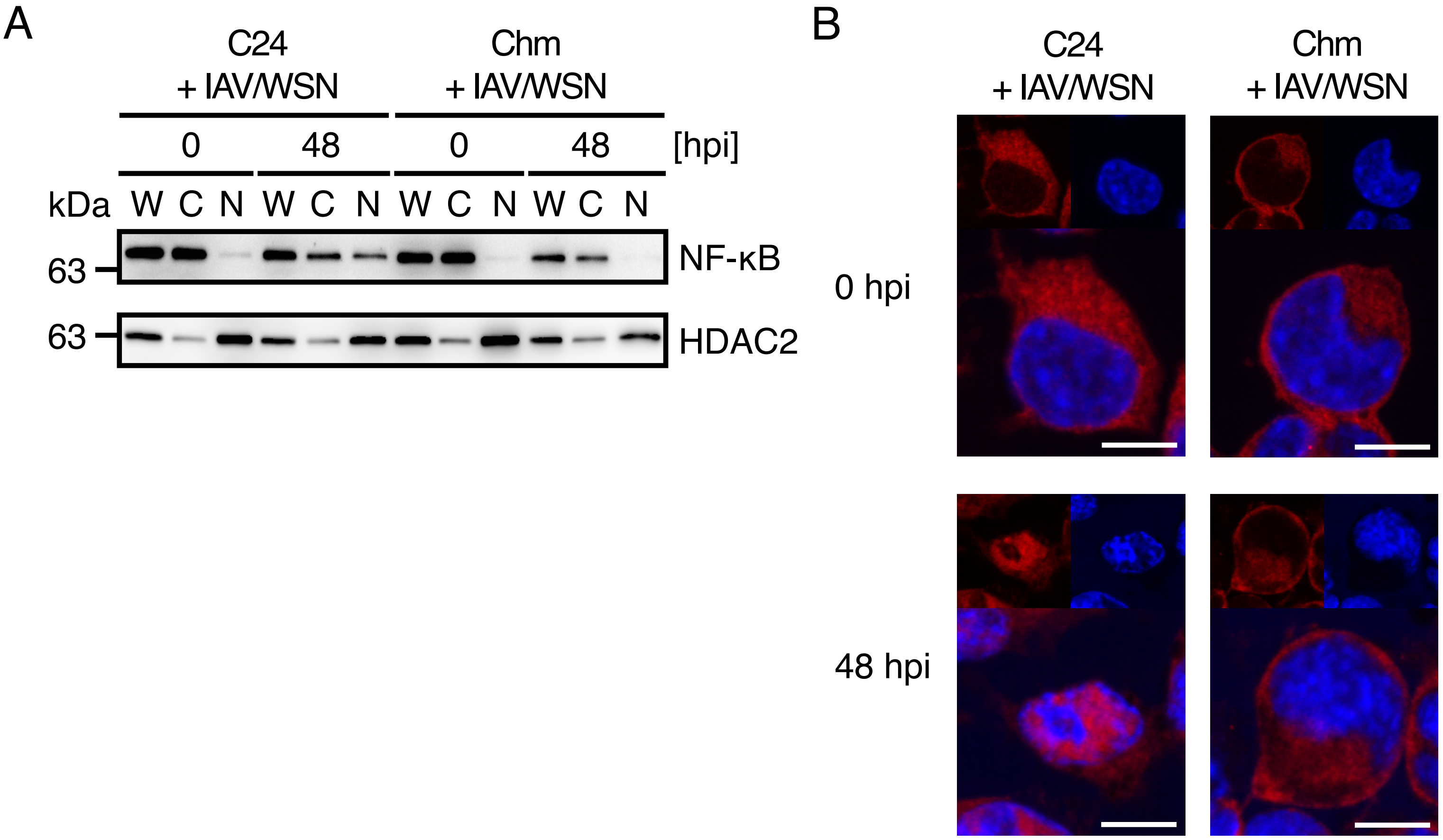

Fig. S4 NF-kB nuclear translocation is disturbed in prion-infected cells after IAV/WSN infection. **(A)** Western blotting for NF-κB p65 in cytoplasmic and nuclear extracts from N2aC24 (C24) and N2aC24Chm (Chm) cells at 0 and 48 hours after IAV/WSN infection. HDAC2 is a nuclear marker. W, whole cell lysate; C, cytoplasmic extract; N nuclear extract. **(B)** Immunofluorescent staining for NF-κB p65 (red) of C24 and Chm cells at 0 and 48 hours after IAV/WSN infection. Blue, DAPI; bar 10 µm. hpi, hours post infection

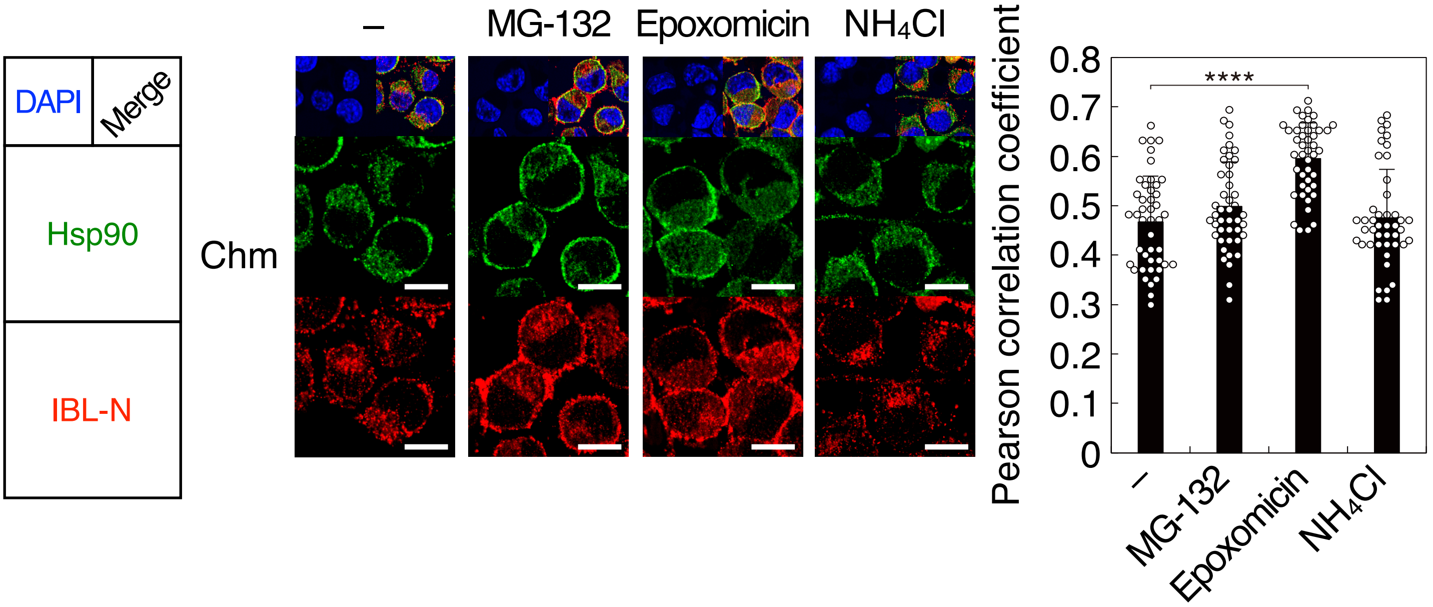

Fig. S5 Portion of the PrP molecule is detectable in the cytoplasm of prion-infected cells. Double immunofluorescent staining for Hsp90 (green) and PrP (red) using anti-HSP90 and IBL-N anti-PrP antibody of N2aC24Chm (Chm) (left panels) cells treated with and without 10 µM MG-132, 1 µM epoxomicin, and 10 mM ammonium chloride. Blue, DAPI; bar 10 µm. Pearson correlation coefficient for the intracellular co-localization of Hsp90 and PrP in Chm (right panel) cells untreated (n = 44) and treated with MG-132 (n = 44), epoxomicin (n = 44), and ammonium chloride (n = 44). ****, $p < 0.001$

Table S1. List of genes, sequences of the primers, and the number of cycles used for RT-PCR gene expression analysis

Gene	Primer	Sequence	Cycles
Sod1	sense	5’- GGG GGA TCC ATG GCG ATG AAA GCG GTG TG -3’	30
	antisense	5’- GGG CTC GAG TTA CTG CGC AAT CCC AAT CA -3’	
Sod2	sense	5’- GGG GGA TCC ATG TTG TGT CGG GCG GCG TG -3’	30
	antisense	5’- GGG CTC GAG TCA CTT CTT GCA AGC TGT GT -3’	
Sod3	sense	5’- GGG GGA TCC ATG TTG GCC TTC TTG TTC TA -3’	30
	antisense	5’- GGG CTC GAG TTAAGT GGT CTT GCA CTC GC -3’	
Gapdh	sense	5’- CAT CAC CAT CTT CCA GGA -3’	20
	antisense	5’- GAG GGG GCC ATC CAC AGT CTT C -3’	
